# Sertoli Leydig Cell Tumour Initially Misdiagnosed as Polycystic Ovarian Syndrome and Congenital Adrenal Hyperplasia: A Case Report

**DOI:** 10.31729/jnma.5045

**Published:** 2020-11-30

**Authors:** Pooja Paudyal, Geeta Gurung, Josie Baral, Nisha Kharel

**Affiliations:** 1 Department of Obstetrics and Gynecology, Tribhuvan University Teaching Hospital (TUTH), Maharajgunj, Kathmandu, Nepal

**Keywords:** *hyperandrogenism*, *Sertoli-Leydig cell tumor*, *tumor differentiation*

## Abstract

Sertoli-Leydig cell tumor of the ovary is an unusual neoplasm that belongs to a group of sex cord-stromal tumors of the ovary and accounts for less than 0.5% of all primary ovarian neoplasms. They are often characterized by the presence of mass with androgen production and signs of virilization. Due to the substantially low incidence of Sertoli-Leydig cell tumors, information on clinical behavior, prognostic factors, and optimal management arelimited. Here in, we report a case of aprimary ovarian Sertoli-Leydig cell tumor in a 21-year-old student, previously diagnosed to have polycystic ovarian syndrome and subsequently congenital adrenal hyperplasia, who presented with a large abdominal mass and features of virilization along with elevated serum testosterone levels. Fertility sparing unilateral salpingo-oophorectomy was done and adjuvant chemotherapy was given after histopathology showed moderate to poorly differentiated Sertoli-Leydig cell tumor. Following surgery, her features of hyperandrogenism resolved and serum testosterone levels returned to normal.

## INTRODUCTION

Sertoli-Leydig cell tumors (SLCTs) are sex cord-stromal tumors that account for less than 0.5% of primary ovarian neoplasms. They are mostly encountered in women in their second to the third decade of life, frequently unilateral, confined to the ovary, and nearly 90% at stage I at the time of diagnosis. These tumors are classically present with features of hyperandrogenism in the form of hirsutism, menstrual irregularities, and clitoromegaly.^1^ Management of SLCTs remains challenging, owing to the lack of standardized management guidelines. Preservation of fertility is an important consideration as they occur predominantly in young girls. Prognosis correlates with the degree of tumor grading and staging.^2^

## CASE REPORT

We report a case of a 21 years student who had a history of irregular cycles with severe dysmenorrhea for three years and had been diagnosed as Polycystic Ovarian Syndrome (PCOS) two years back, after ultrasonography showed a bulky multicystic left ovary, though her hormone profile including testosterone was normal. She was treated with oral contraceptive pills and was under regular follow up since then. For the last six months, the patient also had excessive hair growth for which she was undergoing laser therapy.

On evaluation by an endocrinologist, her total serum testosterone level was elevated to 604.3ng/dl (normal 14-76ng/dl) and 17 hydroxy progesterone was also increased to 10.2ng/ml (normal 0.3-2.5ng/ml). She was then diagnosed to have Congenital Adrenal Hyperplasia (CAH) and was prescribed tablet spironolactone. Her latest ultrasonography showed an increased size of the left ovary, so she was referred to our hospital.

**Figure 1:**
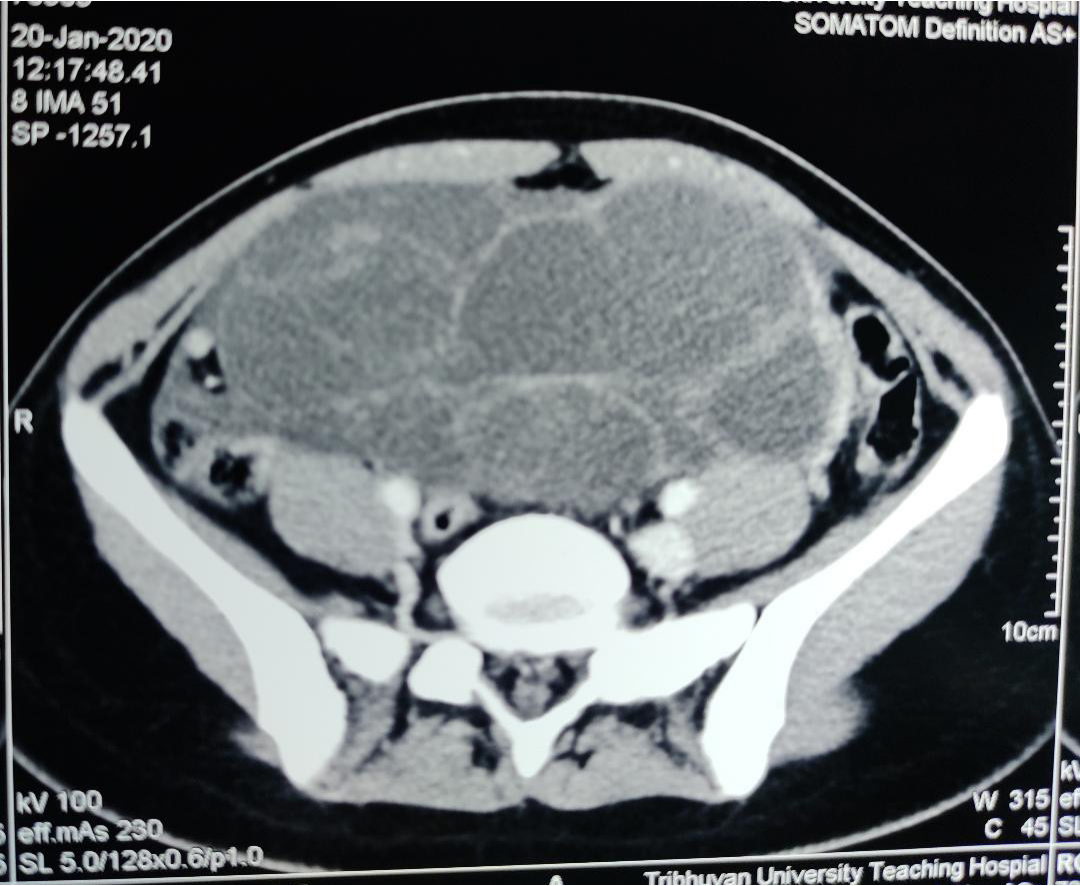
CECT abdomen and pelvis showing a multicystic mass.

On examination, she had hirsutism, well-developed breasts, clitoromegaly, and a palpable mass of 20 cm x15cm in the left iliac fossa and hypogastric region. Contrast-Enhanced Computed Tomography (CECT) showed 17 cm x 17 cm x 11.4 cm well-defined multicystic complex lesion arising from left adnexa, without ascites or lymph node involvement (Figure 1).

**Figure 2:**
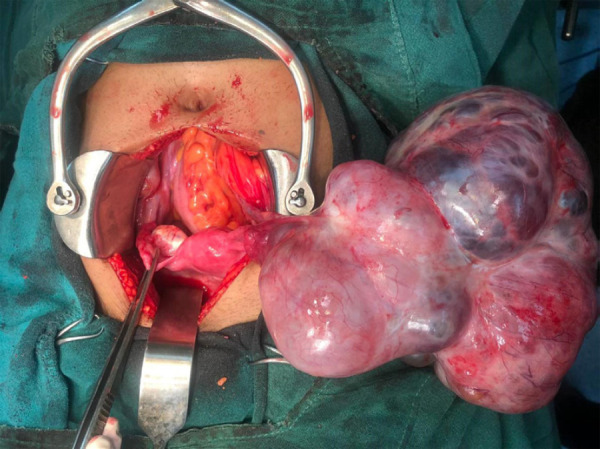
Intraoperative picture showing mass arising from left ovary.

Her tumor markers were normal except Cancer Antigen-125 (CA-125) which was slightly raised to 77.2U/ml (normal <35 U/ml). Based on her clinical and radiological features, a provisional diagnosis of the androgen-secreting ovarian tumor was made and she underwent staging laparotomy with leftsalpingo-oophorectomy with omentectomy.The left ovary was replaced by a 20 cm x17cm irregular multilobulated, solid-cystic mass with an intact capsule. Her contralateral ovary was normal. Minimal ascites were present which was sent for cytology but lymphadenopathy was not noted. The abdominal and pelvic cavities were explored systematically, and there were no macroscopic tumor deposits (Figure 2).

**Figure 3:**
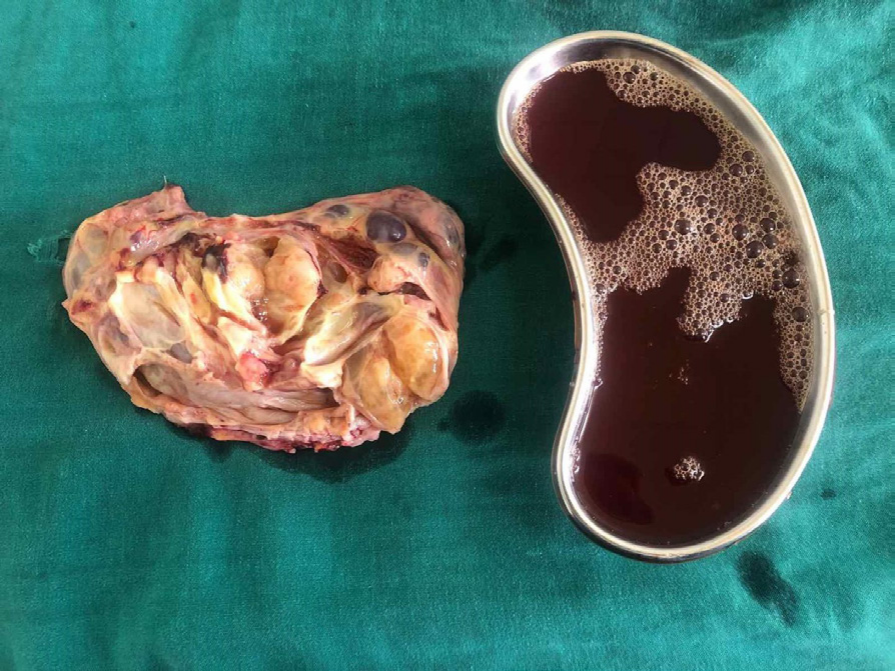
Cut-section of the mass.

The surgical staging was stage 1A. Peritoneal fluid cytology did not reveal any malignant cells. The post-operative period was uneventful and she was discharged on 4th postoperative day (Figure 3).

The histopathology report was sertoli-leydig cell tumor mainly intermediately differentiated (grade 2) and focally poorly differentiated (grade 3) and omentum was free of tumor deposits.

**Figures 4 (A and B):**
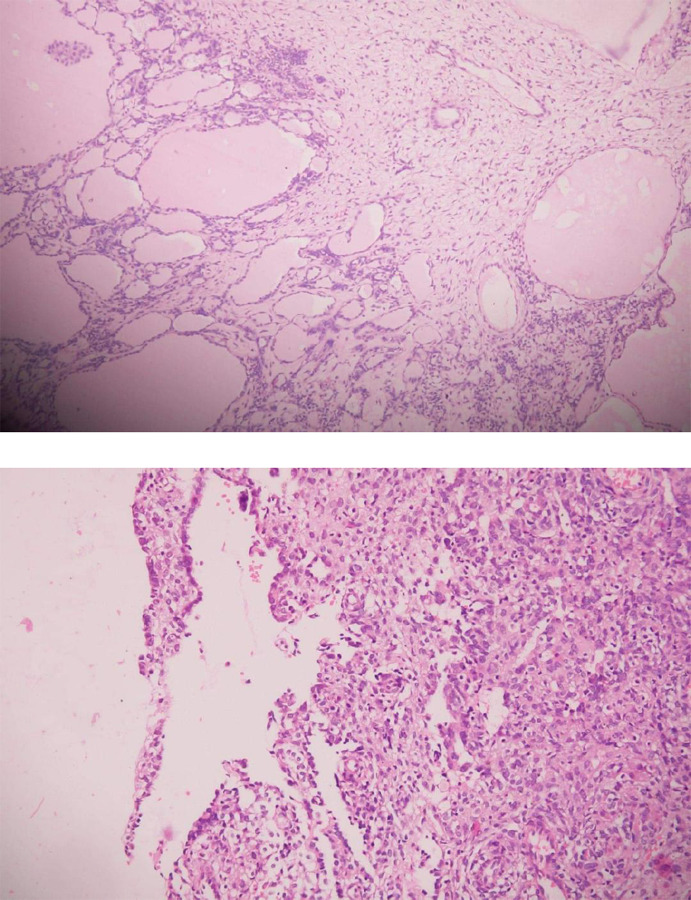
HPE showing tubules lined by Sertoli cells with intervening nests of Leydig cells.

The patient was administered 4 cycles of adjuvant chemotherapy with bleomycin, etoposide, and cisplatin (BEP) regimen which she tolerated well. During follow up at three months, her cycles became regular, hirsutism had improved and serum testosterone level had decreased to 18.9ng/dl.

## DISCUSSION

Sertoli-Leydig cell tumorsare thought to originate from the specialized gonadal stroma and contain Sertoli cells or Leydig cells in varying proportions. They are typically unilateral and confined to the ovary in 97% of cases. They occur in all age groups but are predominant in young women (between the ages of 20 and 30) with an average age of 25 years.^1^ Our patient was 21 years old. Clinical presentation is related to either presence of abdominopelvic mass or hormone production (mostly androgen and rarely estrogen).^3,4^ Clinical manifestation varies widely from an asymptomatic clinical profile to extreme virilization. In one-third of cases, there is the classical presentation with features of progressive masculinization such as hirsutism, temporal balding, deepening of the voice, enlargement of the clitoris, oligomenorrhea, and amenorrhea. These characteristics are due to an increase in androgen production by tumor cells.^1^ Our patient presented initially with irregular cycles along with cystic left ovary and was misdiagnosed as PCOS. Later she had increasing hirsutism and high testosterone levels which was attributed to CAH. The diagnosis of SLCT was made only later when she presented to us with a large rapidly growing mass and signs of virilization consistent with the classical presentation of SLCTs.

SLCTs vary in size from 0.8 to 30 cm with an average of 13.5 cm; are well-encapsulated, solid-cystic, and yellow-gray mass.^4,5^ Microscopically, SLCTs consist of tubules lined by Sertoli cells with intervening nests of Leydig cells with varying degrees of differentiation-well, intermediately, and poorly differentiated and containing heterologouselements.^5^

Management of ovarian SLCTs is challengingdue to the lack of standardized management guidelines owing to their rarity. Surgical removal of the mass remains the goldstandard. Asthey occur predominantlyin young women, preservation of fertilityis an important consideration. Conservative unilateral salpingo-oophorectomy seems to be sufficient and justifiable in a well-differentiated tumor with no evidence ofextension beyond the involved ovary.^1^ In cases of moderate and poorly differentiated SLCTs and those with heterologous elements, hysterectomy with bilateral salpingo-oophorectomyand staging should be performed.^6^ Ours was a young woman with a tumor confined to one ovary hence unilateral salpingo-oophorectomy was deemed sufficient.

Owing to the paucity of randomized clinical trials, information on adjuvant chemotherapy is limited and the mostappropriate treatment regimen and efficacy are still uncertain. Adjuvant chemotherapy isrecommended for patients higher than stage 1, with high mitotic profile, moderatelyor poorly differentiated tumors, or tumors containingheterologous elements and tumor rupture.^4,7,8^ Currently BEP regimen is often used.^2,7,8^ Even though our patient had a surgical stage IA, due to moderate to poor differentiation of tumor, she was treated with adjuvant chemotherapy.

The most important prognostic factors in SLCTs are their stage and degree of differentiation. In a review of 207 cases by Young and Scully, 100% of well-differentiated tumors were benign, whereas 11% with intermediate differentiation, 59% with poor differentiation, and 19% with heterologous elements were malignant.4 In previous studies, the overall 5-year survival rate for well-differentiated SLCTs is reported as 100%, whereas for moderately and poorly differentiated SLCTs is collectively 80%.^4,9^ The overall 5-year survival rate is 95% for stage I and nearlynone for stages III and IV.^4^

These are rare tumors and there could be a diagnostic dilemma if they present with non-classical symptoms. When a young woman with an ovarian tumor and signs of androgen excess presents, the possibility of SLCT should be considered as catching these tumors early is of prognostic significance. Fertility-sparing surgery is the preferred option in young women when diagnosed in early-stage. Although information about chemotherapy is limited, BEP is a commonly used and safe regimen. The poorer degrees of differentiation and advanced staging is related to poor prognosis and mandate long term follow up.
